# Embolização de Artéria Septal com Onyx^®^ na Carmiodiopatia Hipertrófica. Relato de Dois Casos

**DOI:** 10.36660/abc.20240656

**Published:** 2025-06-18

**Authors:** Anna Luiza Souza, Patrícia Ferreira Demuner, Giulliano Gardenghi, Débora Rodrigues, Fernando Henrique Fernandes, Sidney Munhoz, Maurício Lopes Prudente

**Affiliations:** 1 Hospital Encore Aparecida de Goiânia GO Brasil Hospital Encore, Aparecida de Goiânia, GO – Brasil; 2 Laboratório de Hemodinâmica e Cardiologia Intervencionista do Centro-Oeste Cuiabá MT Brasil Laboratório de Hemodinâmica e Cardiologia Intervencionista do Centro-Oeste, Cuiabá, MT – Brasil

**Keywords:** Cardiomiopatia Hipertrófica, Septos Cardíacos, Obstrução da Via de Saída Ventricular Esquerda, Embolização Terapêutica

## Introdução

A cardiomiopatia hipertrófica (CMH) é uma doença de origem genética, hereditária, que se manifesta desde formas assintomáticas até quadros graves, incluindo a morte súbita, com uma incidência anual estimada de 0,5–1%.^1,2^ Os pacientes com obstrução da via de saída do ventrículo esquerdo (VSVE) sintomática podem ser candidatos às terapias de redução septal.^3^ O tratamento cirúrgico é considerado padrão ouro, sobretudo quando há indicação de abordagem valvar no mesmo procedimento. O tratamento percutâneo mostrou resultados clínicos muito próximos quando comparados à cirurgia, como a melhora dos sintomas, a redução do gradiente e da espessura do septo, e a diminuição da regurgitação mitral. Entretanto, na alcoolinização, observou-se maior incidência de implante de marcapasso e de reintervenções, sem impacto na redução da mortalidade.^3,4^

Atualmente, um agente não alcoólico, denominado copolímero de etileno-álcool vinílico (EVOH - Onyx^®^, *Micro Therapeutics*, Inc. ev3 Neurovascular Irvine, CA, EUA) tem sido amplamente utilizado nas intervenções vasculares percutâneas, como na embolização de aneurismas e malformações arteriovenosas com sucesso.^5,6^ Trata-se de um composto que, além do EVOH (Onyx^®^), possui suspensão de pó de tântalo, o que confere nítida radiopacidade e facilita o controle de infusão e do preenchimento do vaso a ser embolizado.^5^ Para garantir a homogeneidade e distribuição uniforme da solução, utilizamos o misturador VortexR (*Scientific Industries*, Bohemia, NY, USA) apropriado para o frasco.

Relatamos dois casos de pacientes com CMH sintomáticos submetidos à embolização septal com EVOH (Onyx^®^).

Esse estudo foi aprovado pelo comitê de ética do Hospital de Urgências de Goiás sob o número CAAE: 81824624.6.0000.0033.

## Relato de Caso 01

Paciente masculino, 58 anos, com CMH septal sintomática, apresentando dispneia aos pequenos esforços (CF III NYHA) apesar da farmacoterapia otimizada, e lipotimia e precordialgia recorrentes. Informou histórico de hipertensão arterial sistêmica, dislipidemia, hipotireoidismo e doença coronariana crônica.

Ao exame físico, encontrava-se estável hemodinamicamente e eupneico em ar ambiente. Ritmo cardíaco regular com sopro sistólico em foco aórtico 2+/6+ e foco mitral 3+/6+.

O ecocardiograma transtorácico (ETT) evidenciou CMH obstrutiva assimétrica (volume de átrio esquerdo 73mL/m^2^, massa do ventrículo esquerdo 458g, septo 22mm; parede posterior 16mm; gradiente de pressão de pico de 117mmHg em repouso) e movimento anterior sistólico (MAS) de valva mitral, gerando insuficiência mitral (IM) moderada e obstrução dinâmica na VSVE. Função sistólica biventricular preservada.

**Figura 1:**
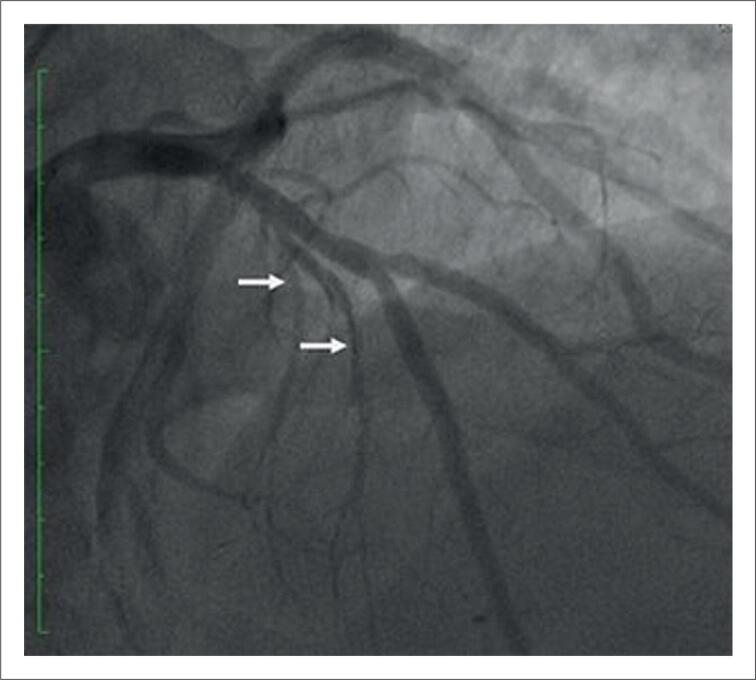
Cineangiocoronariografia pré-operatória com primeiro ramo septal ramificado e calibroso.

A coronariografia revelou artéria descendente anterior (ADA) com lesão de 30%, e o primeiro ramo septal ramificado e calibroso (Figura 1). Na manometria, o gradiente sistólico entre o ventrículo esquerdo e a aorta (VE-AO) era de 80mmHg e o gradiente pós-extrassistólico de 170mmHg.

**Figura 2:**
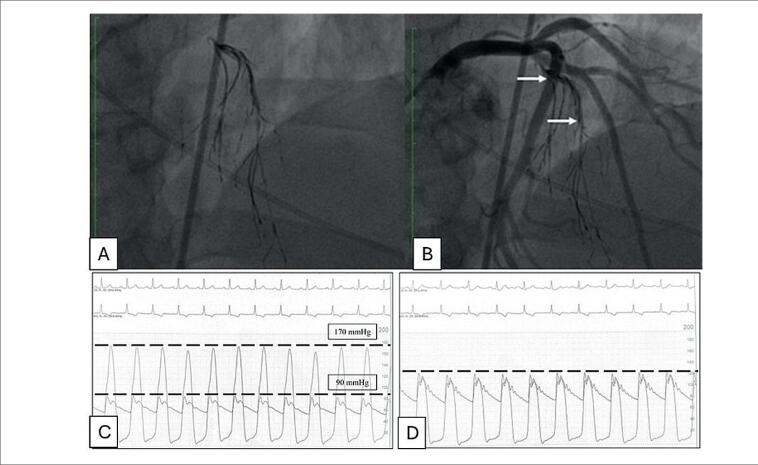
Angiografias de controle. 2A: Radiopacidade permanente do ramo septal após embolização por EVOH (Onyx^®^) confirmando oclusão total do vaso; 2B: Cineangiocoronariografia com septal embolizado e demais artérias pérvias. Manometrias: 2C: Gradiente de pressão entre o ventrículo esquerdo e a aorta de 80mmHg (pré); 2D: Ausência de gradiente após embolização.

Devido à persistência dos sintomas e ao cenário anatômico, optamos pela embolização septal com EVOH (Onyx^®^). O procedimento foi realizado sob sedação e anestesia local, via artéria femoral direita com introdutor 6F (CORDIS AVANTI^®^+) com passagem de cateter guia XB 3.5 6F (Cordis, Warren, NJ, EUA). A artéria radial direita foi puncionada com introdutor 5F radial, com passagem de cateter Pig Tail posicionado na porção apical do ventrículo esquerdo para manometria simultânea com o cateter XB já posicionado na aorta. Após cateterização do tronco da coronária esquerda avançamos o fio-guia Avigo^®^ 0,014" no primeiro ramo septal e através desse o microcateter Echelon^®^ 10", compatível com dimetilsulfóxido (DMSO). Posicionamos um segundo fio-guia 0,014" na porção distal da ADA e através desta um balão oclusor HyperGlide^®^ *Occlusion Balloon System* 4.0x20mm (ambos *Micro Therapeutics*, Inc. ev3 Neurovascular Irvine, CA, USA) ocluindo o segmento da origem do primeiro ramo septal. Este é um balão complacente, usado no território neurovascular; transita sobre fio-guia 0,014" e necessita da presença desse no seu interior para ser insuflado. Realizado teste de oclusão do ramo septal pela insuflação do balão HyperGlide^®^ e injeção de contraste através do microcateter, sem refluxo para ADA e tronco da coronária esquerda, e sem outras conexões com o vaso estudado. Com o objetivo de prevenir a polimerização do EVOH (Onyx^®^) dentro do lúmen do microcateter em contato com o soro ou sangue, injetamos o solvente DMSO, que compõe o kit, com volume exato para preenchê-lo (espaço morto de 0,34mL), imediatamente a seguir injetamos lentamente cerca de 2 a 3 ml do EVOH (Onyx^®^) até total opacificação do ramo septal não permitindo refluxo do mesmo para ADA. Ao final notamos fluxo distal preservado TIMI-III e oclusão total do ramo septal visualizado pela radiopacidade do EVOH (Onyx^®^) (Figura 2A e 2B). A manometria (Figura 2C e 2D) evidenciou resolução completa do gradiente de pressão na VSVE.

O paciente recebeu alta hospitalar após 48 horas. Na consulta de quatro e doze meses, retornou com persistência do gradiente de pico da VSVE de 75mmHg, porção basal do septo de 19mm e afilamento (11mm) com acinesia do terço distal deste mesmo segmento, persistindo com IM moderada por MAS.

Diante da manutenção do gradiente na VSVE e da recidiva dos sintomas (CF III NYHA), repetiu-se o procedimento, após 15 meses da primeira abordagem, com embolização de mais um ramo septal. Nessa ocasião, estava com gradiente na VSVE em sala de 70mmHg (pré-procedimento) e reduziu para 8mmHg (pós-intervenção) além de obter melhora do grau da regurgitação mitral de moderado a importante para leve. O paciente manteve estabilidade elétrica e sem sintomas anginosos. Atualmente está em CF I NYHA.

## Relato de Caso 02

Paciente masculino, 46 anos, com CMH septal assimétrica sintomática, em CF III, a despeito das medicações otimizadas. Ao exame físico, ritmo cardíaco regular com sopro sistólico (foco aórtico 3+/6+ e borda esternal esquerda) e sopro mitral 3+/6+.

**Figura 3:**
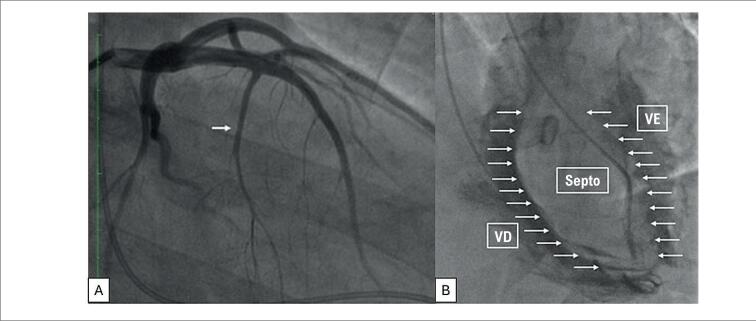
A) Cineangiocoronariografia evidenciando grande ramo septal. B) Hipertrofia septal visualizada por ventriculografia bilateral. VE: ventrículo esquerdo. VD: ventrículo direito.

O ETT mostrou volume de átrio esquerdo de 69 mL/m^2^; massa de ventrículo esquerdo 352g; espessura do septo de 31mm; espessura da parede posterior de 13mm; gradiente de pressão de pico de 114mmHg; gradiente médio de 60,5mmHg, IM moderada por MAS e obstrução dinâmica na VSVE. Função sistólica biventricular normal. Na cineangiocoronariografia, grande ramo septal sem lesões obstrutivas (Figura 3A). Ventriculografia bilateral simultânea evidenciou septo interventricular hipertrófico (Figura 3B).

**Figura 4:**
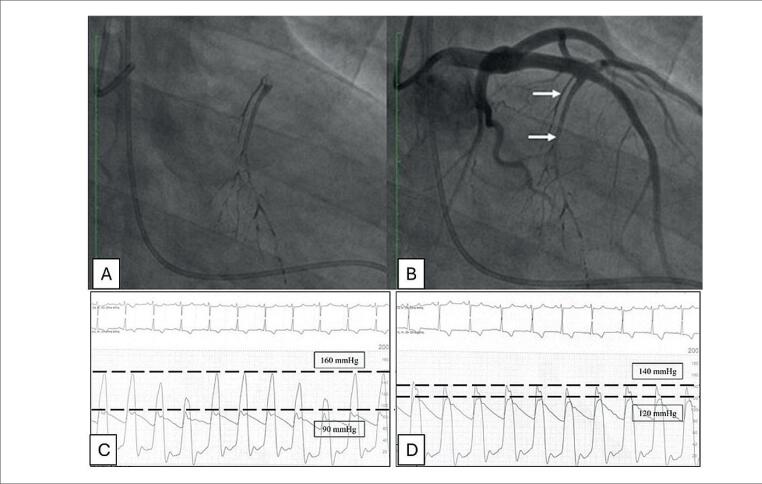
Angiografias. A) Ramo septal radiopaco após embolização por EVOH (Onyx^®^); B) Ramo septal embolizado e demais artérias pérvias; Manometrias. C) Gradiente de pressão entre o ventrículo esquerdo e a aorta de 70mmHg (pré); D) Gradiente após embolização de 20mmHg.

Frente ao quadro, realizamos embolização septal utilizando cerca de 2 a 3mL de EVOH (Onyx^®^) (Figura 4A e 4B). Ao final houve redução do gradiente de pressão da VSVE de 70mmHg para 20mmHg (Figura 4C e 4D).

O paciente recebeu alta hospitalar após 48 horas. Permaneceu assintomático após cinco e 12 meses. ETTs demonstraram gradiente de VSVE de pico de 26mmHg e 45mmHg, respectivamente. Observou-se melhora da IM de moderada para leve. O gradiente da VSVE inicialmente de 80mmHg reduziu para 45mmHg após doze meses e o septo de 31mm caiu para 15mm.

## Discussão

A escolha do tratamento ideal na CMH depende de aspectos clínicos, angiográficos e anatômicos. A miectomia septal, realizada em centro especializado, é a terapia de escolha para os pacientes refratários ao tratamento farmacológico.^7^ No Brasil, existem poucas unidades de referência em miectomia. Devido a isso, torna-se importante desenvolver novas técnicas mais facilmente reprodutíveis e com resultados não inferiores ou melhores em relação à miectomia. A terapia percutânea é recomendada quando há alto risco cardiovascular perioperatório.^4^ Sabe-se que a ablação com álcool resulta em menor tempo de internação e recuperação, embora esteja associada à maior incidência de bloqueios Atrioventriculares (AVs) completos, com necessidade de marcapasso, decorrentes da necrose química não controlada do miocárdio.^7^

Os agentes como cianoacrilato, micromolas de liberação controlada, radiofrequência e cola, são capazes de reduzir o gradiente da VSVE gerando um infarto mais limitado. Entretanto, quando comparados ao álcool, esses agentes não alcoólicos parecem não mostrar os mesmos resultados a longo prazo, devido a recidiva do gradiente na VSVE por retorno ou persistência das alterações fixas (espessura do septo) e dinâmicas (espessamento sistólico e estreitamento desse "túnel" com aceleração do fluxo e MAS), provavelmente por novas colaterais irrigando o septo.^5,6^

O EVOH (Onyx^®^) tem sido implementado na redução septal. A predileção pelo EVOH (Onyx^®^) se dá pela sua propriedade não adesiva, menos trombogênica, capaz de penetrar artérias profundas. Sua segurança baseia-se na maior viscosidade e tempo de polimerização, quando comparado a outros líquidos. Sua radiopacidade notória permite excelente visualização e consequentemente controle da injeção.^5,6^

Em um estudo turco, Osman et al.,^4^ utilizaram o EVOH (Onyx^®^) para embolização septal em 25 pacientes com CMH. Nesse estudo, 8% dos pacientes cursaram com bloqueio AV completo e implante de marcapasso. Em um paciente, observou-se refluxo do EVOH (Onyx^®^) para a artéria diagonal, sem implicações clínicas. Houve necessidade de embolização de duas artérias septais de cinco pacientes por resultados hemodinâmicos não satisfatórios.^4^ Nesse estudo, houve melhora dos sintomas e da CF, contudo, em três pacientes não foi observada redução do gradiente da VSVE após seis meses, a despeito da queda desse gradiente no pós-operatório imediato.^4^ Cabe ressaltar que no caso 01 aqui apresentado, foi necessário um segundo procedimento por persistência do gradiente residual e, no caso 02, observou-se gradiente residual relativamente alto (45mmHg) no seguimento de 12 meses.

Esse estudo possui limitações que precisam ser destacadas. Não foi realizada ressonância magnética para avaliar potenciais áreas de fibrose após as intervenções. Os resultados foram acompanhados pelo ETT, por ser um método não invasivo e mais acessível, estando, portanto, sujeitos ao viés do examinador. Seria ideal haver um seguimento por um período superior a 12 meses, visando investigar eventuais mudanças nos gradientes e na anatomia dos septos, além de se documentar a evolução do quadro clínico dos indivíduos. Ensaios clínicos randomizados comparando o uso de EVOH (Onyx^®^) aos métodos mais tradicionais são necessários. Ainda, há a necessidade de um estudo nacional multicêntrico, visando aumentar a casuística e o tempo de seguimento para caracterização dos achados.

## Conclusão

Nos dois casos de CMH aqui relatados, o uso do EVOH (Onyx^®^) para redução septal mostrou-se uma opção terapêutica viável e que resultou em melhora dos sintomas em pacientes refratários ao tratamento medicamentoso otimizado, com baixo tempo de internação e sem complicações a curto prazo.

## Disponibilidade de Dados

Todo o conjunto de dados que dá suporte aos resultados deste estudo está disponível mediante solicitação ao autor correspondente, considerando a Lei geral de proteção de dados e a preservação da identidade dos participantes da pesquisa.
