# Supplementary material for: Embolização de Artéria Septal com Onyx® na Carmiodiopatia Hipertrófica. Relato de Dois Casos
Source: Arq Bras Cardiol. 2025 Jun 18;122(5):e20240656. [Article in Portuguese] doi: 10.36660/abc.20240656 (PMC12212384; doi:10.36660/abc.20240656)
Supplement: Translation

# Translation: Septal Artery Embolization with Onyx^®^ In Hypertrophic Cardiomyopathy: Report of Two Cases

**DOI:** 10.36660/abc.20240656i

**Authors:** Anna Luiza Souza, Patrícia Ferreira Demuner, Giulliano Gardenghi, Débora Rodrigues, Fernando Henrique Fernandes, Sidney Munhoz, Maurício Lopes Prudente

**Affiliations:** 1 Aparecida de Goiânia GO Brazil Hospital Encore, Aparecida de Goiânia, GO – Brazil; 2 Cuiabá MT Brazil Laboratório de Hemodinâmica e Cardiologia Intervencionista do Centro-Oeste, Cuiabá, MT – Brazil

**Keywords:** Hypertrophic Cardiomyopathy, Heart Septum, Left Ventricular Outflow Obstruction, Therapeutic Embolization

## Introduction

Hypertrophic cardiomyopathy (HCM) is a genetic, hereditary disease that can manifest in asymptomatic forms or severe conditions, including sudden death, with an estimated annual incidence of 0.5–1%.^1,2^ Symptomatic patients with left ventricular outflow tract (LVOT) obstruction may be candidates for septal reduction therapies.^3^ Surgical treatment is considered the gold standard, especially when there is an indication for valvular intervention in the same procedure. Percutaneous treatment has shown clinical results very similar to surgery, such as symptom improvement, gradient and septal thickness reduction, and decreased mitral regurgitation. However, in alcohol septal ablation, a higher incidence of pacemaker implantation and reinterventions was observed, without impact on mortality reduction.^3,4^

Currently, a non-alcoholic agent, known as ethylene-vinyl alcohol copolymer (EVOH - Onyx^®^, Micro Therapeutics, Inc. ev3 Neurovascular Irvine, CA, USA), has been widely used in percutaneous vascular interventions, such as embolization of aneurysms and arteriovenous malformations, with success.^5,6^ This compound, in addition to EVOH (Onyx^®^), contains tantalum powder suspension, which provides clear radiopacity and facilitates infusion control and vessel filling during embolization.^5^ To ensure homogeneity and uniform distribution of the solution, we used the VortexR mixer (Scientific Industries, Bohemia, NY, USA), which is suitable for the vial.

We report two cases of symptomatic HCM patients who underwent septal embolization with EVOH (Onyx^®^).

This study was approved by the ethics committee of Goias Urgent Care Hospital (CAAE: 81824624.6.0000.0033).

## Case Report 01

Male patient, 58 years old, with symptomatic septal HCM, presenting dyspnea on mild exertion (NYHA class III) despite optimized pharmacotherapy, along with recurrent presyncope and precordial pain. He reported a history of systemic arterial hypertension, dyslipidemia, hypothyroidism, and chronic coronary artery disease.

On physical examination, he was hemodynamically stable and eupneic on room air. Regular heart rhythm with a systolic murmur in the aortic (2+/6+) and mitral (3+/6+) areas.

Transthoracic echocardiogram (TTE) revealed asymmetric obstructive HCM (left atrial volume of 73mL/m^2^, left ventricular mass of 458g, septum 22mm; posterior wall 16mm; peak pressure gradient of 117mmHg at rest) and systolic anterior motion (SAM) of the mitral valve, causing moderate mitral insufficiency (MI) and dynamic obstruction in the LVOT. Biventricular systolic function was preserved.

Coronary cineangiography revealed a 30% lesion in the left anterior descending (LAD) artery, and a large, ramified first septal branch (Figure 1). In manometry, the systolic gradient between the left ventricle and the aorta (LV-AO) was 80mmHg, while the post-extrasystolic gradient was 170mmHg.

**Figure 1:** Preoperatory coronary cineangiography showing a large, ramified first septal branch.

Due to the persistence of symptoms and the anatomical situation, we opted for septal embolization with EVOH (Onyx^®^). The procedure was performed under sedation and local anesthesia, via the right femoral artery with a 6F introducer (CORDIS AVANTI^®^+), advancing a XB 3.5 6F guide catheter (Cordis, Warren, NJ, USA). The right radial artery was punctured with a 5F radial introducer, advancing a Pig Tail catheter positioned at the apical portion of the LV for simultaneous manometry with the XB catheter already positioned in the aorta. After catheterization of the left coronary trunk, we advanced the Avigo^®^ 0.014" guidewire into the first septal branch, followed by the Echelon^®^ 10" microcatheter, which is compatible with dimethyl sulfoxide (DMSO). We positioned a second 0.014" guidewire in the distal portion of the LAD, followed by an occlusion balloon, the HyperGlide Occlusion Balloon System 4.0x20mm (both from Micro Therapeutics, Inc. ev3 Neurovascular Irvine, CA, USA), occluding the segment at the origin of the first septal branch. This is a compliant balloon used in the neurovascular territory, which travels over a 0.014" guidewire and requires the presence of the wire inside it to be inflated. A septal branch occlusion test was performed by inflating the HyperGlide balloon and injecting contrast through the microcatheter, with no reflux into the LAD or left coronary trunk, and no other connections to the studied vessel. To prevent the EVOH (Onyx^®^) polymerization inside the microcatheter lumen in contact with serum or blood, we injected the DMSO solvent included in the kit, using the exact volume required to fill it (dead space of 0.34mL). Immediately afterward, we slowly injected approximately 2 to 3 mL of EVOH (Onyx^®^) until complete opacification of the septal branch, preventing reflux into the LAD. At the end, we observed preserved distal flow TIMI-III and total occlusion of the septal branch, visualized by the radiopacity of EVOH (Onyx^®^) (Figures 2A and 2B). Manometry (Figures 2C and 2D) showed complete resolution of the pressure gradient in the LVOT.

**Figure 2:** Control angiographies; A) Radiopacity of the septal branch after embolization with EVOH (Onyx^®^), confirming total vessel occlusion; B) Coronary angiography showing embolized septal branch and patent remaining arteries. Manometry results: C) Pressure gradient between the left ventricle and the aorta of 80mmHg (pre-procedure); D) Absence of gradient after embolization.

The patient was discharged from the hospital after 48 hours. At the four- and twelve-month follow-up visits, he returned with persistent peak LVOT gradient of 75mmHg, basal septal thickness of 19mm, and thinning (11mm) of the distal third of the same segment with akinesia, persisting with moderate MI due to SAM.

Given the persistence of the LVOT gradient and the recurrence of symptoms (NYHA class III), the procedure was repeated 15 months after the initial approach, with embolization of another septal branch. At that time, the LVOT gradient in the procedure room was 70mmHg (pre-procedure) and was reduced to 8mmHg (post-intervention), along with an improvement in the degree of mitral regurgitation from moderate/severe to mild. The patient maintained electrical stability and had no anginal symptoms, and is currently classified as NYHA class I.

## Case Report 02

Male patient, 46 years old, with symptomatic asymmetric septal HCM, classified as NYHA class III, despite optimized medications. On physical examination, the patient had regular heart rhythm with systolic murmur (aortic region 3+/6+ and left sternal border) and mitral murmur 3+/6+.

The TTE showed a left atrial volume of 69 mL/m^2^, left ventricular mass of 352g, septum thickness of 31mm, posterior wall thickness of 13mm, peak pressure gradient of 114mmHg, mean pressure gradient of 60.5mmHg, moderate MI due to SAM, and dynamic obstruction in the LVOT. Biventricular systolic function was normal. In coronary cineangiography, a large septal branch was observed without obstructive lesions (Figure 3A). Simultaneous bilateral ventriculography revealed hypertrophic interventricular septum (Figure 3B).

**Figure 3:** A) Coronary cineangiography showing a large septal branch. B) Septal hypertrophy visualized through bilateral ventriculography; RV: right ventricle; LV: left ventricle.

Given the clinical scenario, we performed septal embolization using approximately 2 to 3mL of EVOH (Onyx^®^) (Figures 4A and 4B). At the end of the procedure, the LVOT pressure gradient was reduced from 70mmHg to 20mmHg (Figures 4C and 4D).

**Figure 4:** Angiographies. A) Radiopaque septal branch after EVOH (Onyx^®^) embolization; B) Embolized septal branch and other patent arteries; Manometry results: C) Pressure gradient between the left ventricle and the aorta of 70mmHg (pre-procedure); D) Pressure gradient after embolization of 20mmHg.

The patient was discharged from the hospital after 48 hours. He remained asymptomatic at five- and 12-month follow-ups. TTEs showed peak LVOT gradients of 26mmHg and 45mmHg, respectively. An improvement in MI was observed, from moderate to mild. The LVOT gradient, initially 80mmHg, decreased to 45mmHg after 12 months, and the septal thickness reduced from 31mm to 15mm.

## Discussion

The choice of the ideal treatment for HCM depends on clinical, angiographic, and anatomical aspects. Septal myectomy, performed in specialized centers, is the treatment of choice for patients who are refractory to pharmacological therapy.^7^ In Brazil, there are few reference centers for myectomy. Therefore, it is important to develop new techniques that are more easily reproducible and yield results that are not inferior or even superior to myectomy. Percutaneous therapy is recommended when there is a high perioperative cardiovascular risk.^4^ It is known that alcohol ablation results in shorter hospitalization and recovery time, although it is associated with a higher incidence of complete atrioventricular (AV) blocks, requiring pacemaker implantation due to uncontrolled chemical necrosis of the myocardium.^7^

Agents such as cyanoacrylate, controlled release microspheres, radiofrequency, and glue can reduce the LVOT gradient, resulting in a more limited infarction. However, when compared to alcohol, these non-alcoholic agents seem to show less favorable long-term results due to the recurrence of the LVOT gradient, either from the return or persistence of fixed (septal thickness) and dynamic (systolic thickening and narrowing of this "tunnel" with flow acceleration and SAM) alterations, likely due to new collateral vessels irrigating the septum.^5,6^

EVOH (Onyx^®^) has been implemented in septal reduction. The preference for EVOH (Onyx^®^) is due to its non-adhesive property, lower thrombogenicity, and ability to penetrate deep arteries. Its safety is based on higher viscosity and polymerization time compared to other liquids. Its remarkable radiopacity allows excellent visualization and, consequently, precise injection control.^5,6^

In a Turkish study, Osman et al.^4^ used EVOH (Onyx^®^) for septal embolization in 25 patients with HCM. In this study, 8% of patients developed complete AV block and required pacemaker implantation. In one patient, reflux of EVOH (Onyx^®^) into the diagonal artery was observed, without clinical implications. It was necessary to embolize two septal arteries in five patients due to unsatisfactory hemodynamic results.^4^ This study showed improvement in symptoms and functional class; however, in three patients, no reduction in the LVOT gradient was observed after six months, despite an immediate postoperative decrease in the gradient.^4^ It is worth noting that in Case 01 presented here, a second procedure was required due to the persistence of residual gradient, and in Case 02, a relatively high residual gradient (45mmHg) was observed at the 12-month follow-up.

This study has limitations that need to be highlighted. Magnetic resonance imaging was not performed to assess potential areas of fibrosis after the interventions. The results were monitored using TTE, as it is a non-invasive and more accessible method, and therefore subject to examiner bias. Ideally, follow-up should extend beyond 12 months to investigate possible changes in gradients and septal anatomy, as well as to document the clinical course of the individuals. Randomized clinical trials comparing the use of EVOH (Onyx^®^) with more traditional methods are necessary. Additionally, a national multicenter study is needed to increase the sample size and extend the follow-up period for better characterization of findings.

## Conclusion

In the two reported cases of HCM, the use of EVOH (Onyx^®^) for septal reduction was shown to be a viable therapeutic option, resulting in symptom improvement in patients refractory to optimized drug treatment, with a short hospitalization period and no short-term complications.

## Data Availability

All datasets supporting the results of this study are available upon request from the corresponding author, considering the General Data Protection Law and the preservation of the identity of the research participants.
